# Hippocampal serotonin depletion unmasks differences in the hyperlocomotor effects of phencyclidine and MK-801: quantitative versus qualitative analyses

**DOI:** 10.3389/fphar.2013.00109

**Published:** 2013-08-29

**Authors:** Wendy K. Adams, Adam L. Halberstadt, Maarten van den Buuse

**Affiliations:** ^1^Behavioural Neuroscience Laboratory, Florey Institute for Neuroscience and Mental Health, University of MelbourneMelbourne, VIC, Australia; ^2^Centre for Neuroscience, University of MelbourneMelbourne, VIC, Australia; ^3^Department of Psychiatry, University of California San DiegoLa Jolla, CA, USA; ^4^Department of Pharmacology, University of MelbourneMelbourne, VIC, Australia

**Keywords:** serotonin, hippocampus, phencyclidine, MK-801, 5,7-dihydroxytryptamine, locomotor hyperactivity, spatial d, entropy

## Abstract

Antagonism of *N*-methyl-D-aspartate (NMDA) receptors by phencyclidine (PCP) is thought to underlie its ability to induce a schizophrenia-like syndrome in humans, yet evidence indicates it has a broader pharmacological profile. Our previous lesion studies highlighted a role for serotonergic projections from the median, but not dorsal, raphe nucleus in mediating the hyperlocomotor effects of PCP, without changing the action of the more selective NMDA receptor antagonist, MK-801. Here we compared locomotor responses to PCP and MK-801 in rats that were administered 5,7-dihydroxytryptamine (5,7-DHT) into either the dorsal or ventral hippocampus, which are preferentially innervated by median and dorsal raphe, respectively. Dorsal hippocampus lesions potentiated PCP-induced hyperlocomotion (0.5, 2.5 mg/kg), but not the effect of MK-801 (0.1 mg/kg). Ventral hippocampus lesions did not alter the hyperlocomotion elicited by either compound. Given that PCP and MK-801 may induce different spatiotemporal patterns of locomotor behavior, together with the known role of the dorsal hippocampus in spatial processing, we also assessed whether the 5,7-DHT-lesions caused any qualitative differences in locomotor responses. Treatment with PCP or MK-801 increased the smoothness of the path traveled (reduced spatial d) and decreased the predictability of locomotor patterns within the chambers (increased entropy). 5,7-DHT-lesions of the dorsal hippocampus did not alter the effects of PCP on spatial d or entropy – despite potentiating total distance moved – but caused a slight reduction in levels of MK-801-induced entropy. Taken together, serotonergic lesions targeting the dorsal hippocampus unmask a functional differentiation of the hyperlocomotor effects of PCP and MK-801. These findings have implications for studies utilizing NMDA receptor antagonists in modeling glutamatergic dysfunction in schizophrenia.

## INTRODUCTION

Phencyclidine (PCP) and MK-801 are often used interchangeably in the psychopharmacological literature as they are both non-competitive antagonists of the glutamatergic *N*-methyl-D-aspartate (NMDA) receptor. It is well-established, however, that MK-801 is more potent at this receptor site than PCP, and that both agents have direct, and dissimilar, effects on other neurotransmitter systems (Lodge and Johnson, 1990; Morris et al., 2005). For example, PCP is a more potent catecholaminergic reuptake inhibitor than MK-801 (Snell et al., 1988; Hiramatsu et al., 1989) and is reported to block reuptake at the serotonin transporter (Hiramatsu et al., 1989; Rothman, 1994; Millan et al., 1999). More recent *in vitro* binding studies distinguish PCP and MK-801 by their relative affinities to the dopamine D_2_ receptor (Kapur and Seeman, 2002; Seeman et al., 2005), although negative findings have also been reported (Millan et al., 1999; Jordan et al., 2006). It is also suggested that PCP has moderate affinity for the serotonin 5-HT_2A_ receptor (Kapur and Seeman, 2002) but this has not been replicated by other studies (Millan et al., 1999; Rabin et al., 2000).

The functional mechanism of action of PCP and its analog, ketamine, is of great interest as they are able to evoke a syndrome in humans resembling the spectrum of symptoms in schizophrenia. These “dissociative anesthetics” are thus distinct from psychostimulants like amphetamine, as they can induce not only positive symptoms but also the negative symptoms and cognitive deficits characteristic of the illness (Luby et al., 1959; Krystal et al., 1994; Halberstadt, 1995; Jentsch and Roth, 1999; Morris et al., 2005). Moreover, while psychostimulants typically require chronic use to elicit psychotic states in healthy subjects, a single dose of a PCP or ketamine can induce schizophrenia-like behavioral disturbances (Abi-Saab et al., 1998; Moghaddam and Jackson, 2003; Stone and Pilowsky, 2006). Indeed, their pharmacological characterisation as NMDA receptor antagonists in the 1980s (Anis et al., 1983; Lodge and Johnson, 1990) led to the development of the “NMDA receptor hypofunction hypothesis of schizophrenia,” which suggests that dopaminergic dysfunction may be secondary to a primary glutamatergic deficit (Jentsch and Roth, 1999; Olney et al., 1999; Svensson, 2000; Javitt, 2004; Coyle, 2006). The ubiquitous distribution of glutamatergic neurons in the brain, and their regulation by neuromodulatory transmitters, make them a likely candidate for dysfunction in schizophrenia. Interactions between the glutamate system and the dopamine or serotonin systems have been widely studied in this context (Aghajanian and Marek, 2000; Svensson, 2000; Coyle, 2006; Meltzer et al., 2011). However, while PCP may be an appropriate pharmacological tool used in modeling the disorder (Lipska and Weinberger, 2000; Morris et al., 2005), whether its schizophrenia-like effects are due entirely to NMDA receptor antagonism remains to be determined (Kapur and Seeman, 2002; Seeman et al., 2005; Seeman and Lasaga, 2005).

Previous studies have provided evidence of differential serotonergic involvement in the hyperlocomotor effects of PCP and MK-801. For example, PCP-induced locomotor behavior in rats is attenuated by the administration of 5-HT_2A_ receptor antagonists (Maurel-Remy et al., 1995; Krebs-Thomson et al., 1998; Millan et al., 1999). In contrast, 5-HT_2A_ receptor blockade has less consistent effects on MK-801-elicited hyperactivity (Maj et al., 1996; Higgins et al., 2003), suggesting subtle differences in the mechanism of action of these NMDA receptor antagonists. Indeed, when administered alone, locomotor behaviors such as forward ambulation and stereotypic movements induced by PCP and MK-801 are qualitatively different (Hiramatsu et al., 1989; Tricklebank et al., 1989; Lehmann-Masten and Geyer, 1991; Danysz et al., 1994; Ogren and Goldstein, 1994; Gilmour et al., 2009). Some suggest that this is mediated by the ability of PCP to increase serotonin turnover (Hiramatsu et al., 1989), yet others have reported that MK-801 alters serotonin turnover but not PCP (Martin et al., 1998b). Both PCP- and MK-801-induced locomotor hyperactivity, however, is enhanced by pre-treatment with a 5-HT_2C_ receptor antagonist (Hutson et al., 2000). In fact, the 5-HT_2C_ receptor is emerging as a key serotonin receptor subtype involved in the modulation of locomotor behaviors (Takahashi et al., 2001; Giorgetti and Tecott, 2004; Halberstadt et al., 2009). Serotonergic projections to the hippocampus, in particular, are implicated in the modulation of locomotion (Takahashi et al., 2000; Kusljic and van den Buuse, 2004; Dias Soares et al., 2007) and the 5-HT_2_ receptor family seems especially involved in this region (Takahashi et al., 2001; Dave et al., 2004).

We have extensively studied the role of brain serotonin in models of schizophrenia in rats using the approach of selective lesions. Injection of the serotonergic neurotoxin, 5,7-dihydroxytryptamine (5,7-DHT), into the median, but not the dorsal, raphe nucleus (MnR, DR) was found to potentiate PCP-induced locomotor behaviors (Kusljic et al., 2003, 2005), but not the effect of MK-801 (Kusljic et al., 2005), providing evidence of a pharmacological distinction between these drugs at the level of serotonergic projections originating in the MnR. Local 5,7-DHT administration into MnR projection regions revealed that lesions of the dorsal, but not ventral, hippocampus enhanced both PCP- and ketamine-induced hyperlocomotion (Kusljic and van den Buuse, 2004; Adams et al., 2009). Taken together, these findings raised questions about both the selectivity and sensitivity of lesion effects. Specifically, we wanted to clarify: (1) whether 5,7-DHT-lesions of the dorsal hippocampus are sufficient to distinguish between the actions of PCP and MK-801, like MnR lesions; and (2) whether the lesions also enhance locomotor responses to PCP at a five-fold lower dose. To this end, our first experiment investigated both dorsal and ventral hippocampal lesion effects on locomotor hyperactivity induced by 0.5 and 2.5 mg/kg of PCP or 0.1 mg/kg of MK-801.

In addition, we wished to examine more qualitative aspects of locomotor behavior using a novel method of analyses. Previously, such high resolution approaches have shown that PCP and MK-801 induce different spatiotemporal patterns of locomotor behavior (Lehmann-Masten and Geyer, 1991), and that pre-treatment with serotonin receptor ligands modulates the type of patterns elicited by PCP, creating an entirely new behavioral profile (Krebs-Thomson et al., 1998). Therefore, we conducted a second experiment focusing on more qualitative aspects of locomotor responses to PCP (2.5 mg/kg) and MK-801 (0.1 mg/kg) in dorsal hippocampus lesioned rats, including the “smoothness” and “predictability” of locomotor paths. Given the prominent role of the dorsal hippocampus in spatial information processing, with visuospatial inputs directed mainly to the dorsal, but not ventral, domain (Moser and Moser, 1998; Small, 2002; Bast, 2007), we anticipated our lesions might modulate such spatial aspects of locomotor behavior, either at baseline or following drug treatment.

## MATERIALS AND METHODS

### ANIMALS

Sixty four male Sprague-Dawley rats (aged 4–5 weeks) were obtained from the Department of Pathology, University of Melbourne (Parkville, VIC, Australia), or Monash Animal Services (Clayton, VIC, Australia). Colony conditions were standardized, with a 12/12 h light/dark cycle (lights on 7:00–19:00) and the temperature maintained at approximately 22°C. All procedures were performed in the light phase. Rats were housed in groups of 2–3 in cages enriched with shredded paper and cardboard boxes, with standard food and tap water available *ad libitum*. All surgical and experimental protocols were approved by the Animal Experimentation Ethics Committee of the University of Melbourne or the Howard Florey Institute (Parkville, VIC, Australia), and adhered to the guidelines outlined in the Australian Code of Practise for the Care and Use of Animals for Scientific Purposes (National Health and Medical Research Council of Australia, 2004).

### DRUGS AND SOLUTIONS

To prevent oxidation, 5,7-DHT (5,7-dihydroxytryptamine creatinine sulfate salt, Fluka BioChemika, Sigma-Aldrich, St. Louis, MO, USA) was dissolved in 0.9% saline containing 0.1% ascorbic acid. The selective noradrenaline reuptake inhibitor, desmethylimipramine hydrochloride (DMI; Sigma-Aldrich), was prepared in distilled water and dissolved by sonication. The non-steroidal anti-inflammatory agent, Carprofen (Rimadyl®, 50 mg/ml, Pfizer, West Ryde, NSW, Australia) was diluted in 0.9% saline. Consistent with previous work in our laboratory, PCP hydrochloride (Experiment 1: Sigma-Aldrich; Experiment 2: National Measurement Institute, Pymble, NSW, Australia) and (+)-MK-801 hydrogen maleate (dizocilpine, Sigma-Aldrich) were dissolved in 0.9% saline and administered subcutaneous (s.c.). All doses were taken as the weight of the salt and injection volume was 1 mg/kg body weight.

### STEREOTAXIC LESION SURGERY

Surgery was conducted when animals were 7–8 weeks old as described previously (Kusljic and van den Buuse, 2004; Adams et al., 2008, 2009; Adams and van den Buuse, 2011). In brief, rats were randomly allocated to one of four groups: dorsal hippocampus-injected (DHI), ventral hippocampus-injected (VHI) or their equivalent sham-operated controls. At the outset of surgery, 30 min prior to 5,7-DHT infusion, DMI (20 mg/kg, i.p.) was injected to prevent the destruction of noradrenergic neurons. Animals were then anesthetized using a 10% isoflurane/oxygen mixture and transferred to a stereotaxic apparatus affixed with a nose cone to maintain anesthesia. Carprofen (5 mg/kg, s.c.) was used to minimize post-operative discomfort. Holes were drilled in the skull above the dorsal or ventral hippocampus, using the following coordinates relative to bregma: posterior -3.6 mm, lateral ±1.5 and ±3.5 mm, and ventral -3.8 mm for the dorsal hippocampus; posterior -5.6 mm, lateral ±4.8 mm and ventral -8.0 mm for the ventral hippocampus (Paxinos and Watson, 1998). 5,7-DHT (1 μl, 5 μg/μl) was injected bilaterally into the dorsal or ventral hippocampus over 2 min; for the DHI infusions, two 0.5 μl injections were used. Sham-operated rats received equivalent volumes of vehicle solution. Rats were allowed two weeks to recover from surgery before behavioral experiments started.

### LOCOMOTOR HYPERACTIVITY TESTING

Locomotor activity was measured in eight automated photocell cages (43 cm × 43 cm × 31 cm, l × w × h, ENV-520, Med Associates Inc., St. Albans, VT, USA). Each cage had 16 evenly-spaced infrared transmitters and receivers on each of its four sides, which detected a rat’s position in three dimensions (*x, y* and *z*). Software (Activity Monitor 4.0, Med Associates Inc.) recorded the status of the infrared beams every 50 ms, effectively generating a spatio-temporal map of an animal’s movement throughout a testing session. Every 5 min, the software calculated the total distance moved from these data, reflecting the gross distance traveled by an animal with small repetitive movements filtered out.

Each session included a random allocation of 5,7-DHT-lesioned rats and sham-operated controls. Baseline locomotor activity was initially recorded for 30 min, allowing the animals to habituate to the cages before receiving drug treatment. Post-injection locomotor activity was then recorded for further 90 min. Testing sessions were separated by 3–4 days to allow for drug clearance, and the order of treatment in each experiment was pseudo-randomized to offset potential interactions, such as sensitisation, that could occur between treatments. Baseline activity differences were assessed by averaging pre-injection distance moved across all testing sessions within each experiment. As baseline activity was unaffected by the serotonergic lesions in both experiments, these data were subsequently removed from analyses of drug effects.

### ENZYME-LINKED IMMUNOSORBENT ASSAY (ELISA)

Lesions were confirmed by measuring serotonin levels in the dorsal and ventral hippocampus using Serotonin ELISA kits (Labor Diagnostika Nord GmbH & Co. KG, Nordhorn, Germany), with minor adjustments as described before (Adams et al., 2009; Adams and van den Buuse, 2011). Rats were decapitated at least three days after the end of locomotor testing, and the dorsal and ventral hippocampi dissected out, weighed, and stored at -80°C until ELISA. Serotonin levels were normalized for tissue wet weight. Analyses of ELISA data from DHI- and VHI-sham-operated rats in each experiment found no differences in hippocampal serotonin levels between the two types of controls; therefore, these groups were combined. In line with our previous work (Adams and van den Buuse, 2011), DHI or VHI rats were excluded if the percentage serotonin depletion was <20% in the relevant hippocampal domain compared to sham-operated animals; presently, only one VHI rat was excluded from Experiment 1.

### DESIGN AND ANALYSES

Animals were used in two experimental cohorts. Experiment 1 contained DHI (*n* = 16), VHI (*n* = 8) and equivalent sham-operated controls (*n* = 17); all rats received saline, 0.5 and 2.5 mg/kg PCP, and 0.1 mg/kg MK-801 in locomotor activity tests. Experiment 2 contained only DHI (*n* = 12) and DHI-sham-operated (*n* = 11) rats; all animals received saline, 2.5 mg/kg PCP and 0.1 mg/kg MK-801 in locomotor activity tests; 0.02 mg/kg MK-801 was also tested in these animals yet these data are not presented as this dose had negligible effect on locomotor responses. Using a within-subjects design to assess drug responses was important for comparing the effects of PCP and MK-801 by minimizing the variation that is inherent in between-group comparisons; in addition, it greatly reduced the number of animals required.

In Experiment 1, distance moved data were calculated by the activity monitor software. In Experiment 2, raw *x*, *y*, *t* data were extracted from the software as ASCII text files and analyzed for qualitative aspects of locomotor activity. Analysis of the spatial structure of locomotor paths was performed by calculating the descriptive statistic, spatial d. As described by Paulus and Geyer (1991), spatial d is based conceptually on fractal geometry and calculated using scaling arguments. Changes in d reflect smoother (reduced d values) or rougher (increased d values) locomotor paths. Entropy was used to quantify the predictability of locomotor paths, specifically the predictability of sequences of transitions across different zones of the test chamber (Paulus and Geyer, 1993). For example, a rat repeatedly circling along the outer edges of the chamber would move through zones 1, 2, 3, 6, 9, 4, and back to 1; repetition of this sequence many times would result in a low entropy measure. By contrast, a rat that moves through different zones of the chamber via more diverse routes would result in higher entropy levels.

Analysis of variance (ANOVA) with repeated measures, as appropriate, was used to compare differences between and within groups using SYSTAT software (SYSTAT 9.0, SPSS Inc., Chicago, IL, USA). All data were analyzed separately, initially in an overall ANOVA with data from each group (Experiment 1: sham-operated, DHI, VHI; Experiment 2: sham-operated, DHI). When significant main effects of, or interactions with, group were found in the main analyses, planned (a priori) ANOVA comparisons ensued, with either DHI or VHI groups compared to controls, and drug effects compared to that of saline injection.

Distance moved data from Experiment 1 were analyzed in 5-min intervals to assess possible time-dependent drug effects, whereas distance moved, spatial d and entropy data from Experiment 2 were assessed in 30-min intervals. Post-injection spatial d and entropy data were analyzed for 60 min as low activity levels in the 60–90 min block following saline treatment rendered measurement of these variables unreliable. Thus, overall ANOVAs of all post-injection activity data contained the repeated measures variables, “drug” (Experiment 1: four saline/drug treatments; Experiment 2: three saline/drug treatments) and “time” (Experiment 1: 18 5-min intervals; Experiment 2: two or three 30-min intervals).

Enzyme-linked immunosorbent assay data were analyzed by comparing absolute serotonin levels in either the dorsal or ventral hippocampus. Data are presented as percentage depletion relative to sham-operated animals to control for inter-assay variability.

Differences were considered significant if *p* < 0.05.

## RESULTS

### SEROTONIN DEPLETION IN THE DORSAL AND VENTRAL HIPPOCAMPUS

In Experiment 1, DHI rats showed a comparable level of serotonin depletion in the ventral hippocampus as VHI rats, but a greater extent of depletion in the dorsal hippocampus (**Table 1**). VHI rats in this cohort also showed a slight, but significant, depletion of serotonin in the dorsal hippocampus. Like those in Experiment 1, DHI rats in Experiment 2 showed serotonin depletion in both the dorsal and ventral hippocampus compared to sham-operated controls (**Table 1**).

**Table 1:** Serotonin depletion pattern in the dorsal and ventral hippocampus of 5,7-DHT-lesioned rats.

	Dorsal hippocampus		Ventral hippocampus	
	% Depletion	*F*, *p*	% Depletion	*F*, *p*
**Experiment 1**				
DHI	77 ± 4**	177.0, <0.001	54 ± 5**	34.3, <0.001
VHI	19 ± 8*	5.4, 0.029	45 ± 8*	14.0, 0.001
**Experiment 2**
DHI	79 ± 5**	130.1, <0.001	82 ± 2**	138.1, <0.001

*Data are expressed as mean percentage depletion of serotonin ± SEM in each hippocampal domain relative to the sham-operated controls in each experiment. Statistical results in adjacent columns refer to the *F*- and *p*-values obtained in ANOVA comparison of absolute serotonin levels.*

* p < 0.05

** p < 0.001 compared to sham-operated controls.

### EXPERIMENT 1: LESION EFFECTS ON LOCOMOTOR HYPERACTIVITY INDUCED BY 0.5 AND 2.5 mg/kg PCP AND 0.1 mg/kg MK-801

**FIGURE 1:**
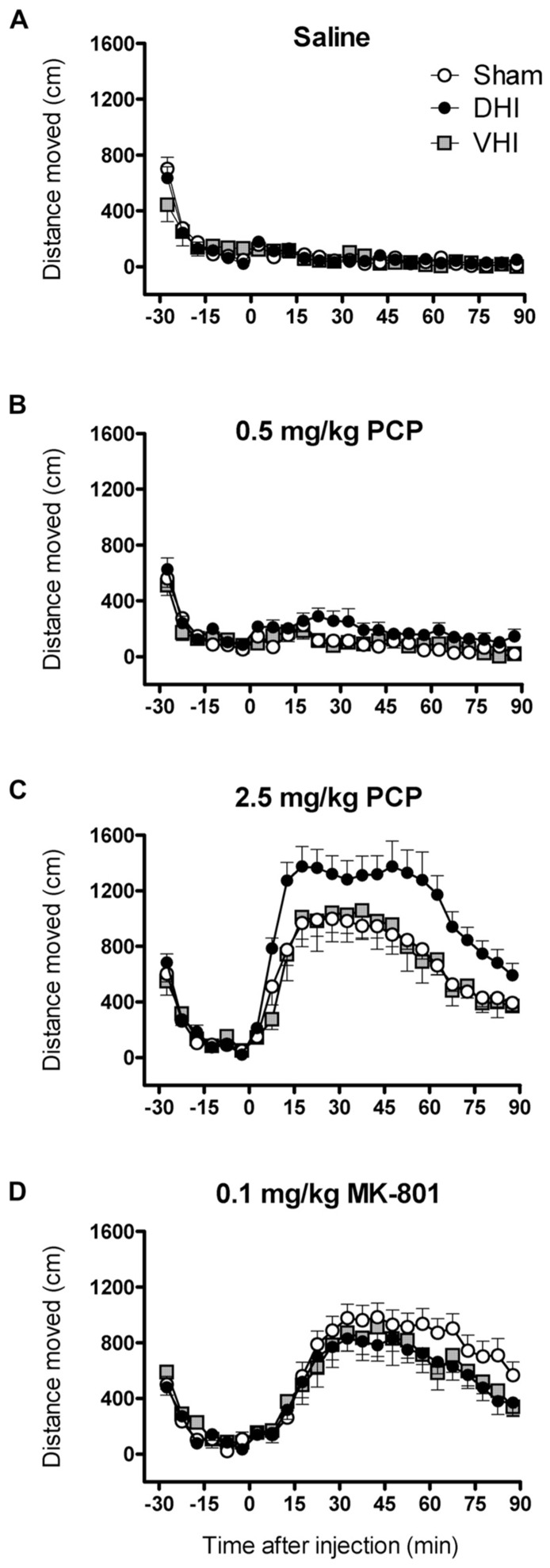
**Effect of 5,7-DHT-lesions targeting the dorsal or ventral hippocampus on locomotor hyperactivity induced by 0.5 and 2.5 mg/kg PCP and 0.1 mg/kg MK-801.** Panels show mean distance moved (cm) in 5 min intervals ± SEM for DHI (*n* = 16), VHI (*n* = 8) and sham-operated rats (*n* = 17) treated with **(A)** saline **(B)** 0.5 and **(C)** 2.5 mg/kg PCP and **(D)** 0.1 mg/kg MK-801. Total distance moved following 0.5 and 2.5 mg/kg PCP injection was significantly higher in DHI rats compared to controls (see text for details).

Analysis of variance of average pre-injection distance moved found no group differences, indicating that 5,7-DHT administration into the dorsal or ventral hippocampus did not affect levels of baseline activity (**Figure 1**). Locomotor activity diminished over this 30 min pre-injection habituation period similarly in all animals (main effect of time: *F*_5,190_ = 163.5, *p* < 0.001).

Analysis of all post-injection distance moved data revealed significant lesion effects (main effect of group: *F*_2,38_ = 3.7, *p*=0.034; drug by group interaction: *F*_6,114_ = 3.7, *p* = 0.002; **Figure 1**). Rats that were administered 5,7-DHT into the dorsal hippocampus showed enhanced locomotor hyperactivity following 0.5 mg/kg PCP treatment, with a 109 ± 37% increase in total post-injection distance moved compared to controls (main effect of group: *F*_1,31_ = 7.2, *p* = 0.012; drug by group interaction: *F*_1,31_ = 6.0, *p* = 0.020; **Figure 1B**). Confirming our previous findings (Kusljic and van den Buuse, 2004), DHI rats also showed potentiated hyperlocomotor effects of 2.5 mg/kg PCP treatment (51 ± 15% increase in total distance moved; main effect of group: *F*_1,31_ = 10.6, *p* = 0.003; drug by group interaction: *F*_1,31_ = 9.3, *p* = 0.005; **Figure 1C**). The enhancement of PCP-induced hyperlocomotion was uniform throughout the session for both doses (lack of significant interactions with time and group; **Figures 1B,C**). In contrast, VHI and sham-operated animals showed similar PCP-induced locomotor hyperactivity at both doses (main effects of drug: 0.5 mg/kg, *F*_1,23_ = 13.4, *p* = 0.001; 2.5 mg/kg, *F*_1,23_ = 133.3, *p* < 0.001; **Figures 1B,C**). Notably, treatment with MK-801 evoked locomotor hyperactivity to a similar extent and temporal magnitude in sham-operated, DHI and VHI rats (main effect of drug: *F*_1,38_ = 127.3, *p* < 0.001; drug by time interaction: *F*_17,646_ = 42.3, *p* < 0.001), indicating that the potentiated hyperlocomotor effect in DHI rats is unique to PCP (**Figure 1D**).

### EXPERIMENT 2: FURTHER ANALYSIS OF DORSAL HIPPOCAMPUS LESION EFFECTS ON LOCOMOTOR HYPERACTIVITY INDUCED BY 2.5 mg/kg PCP AND 0.1 mg/kg MK-801

#### Distance moved

**FIGURE 2:**
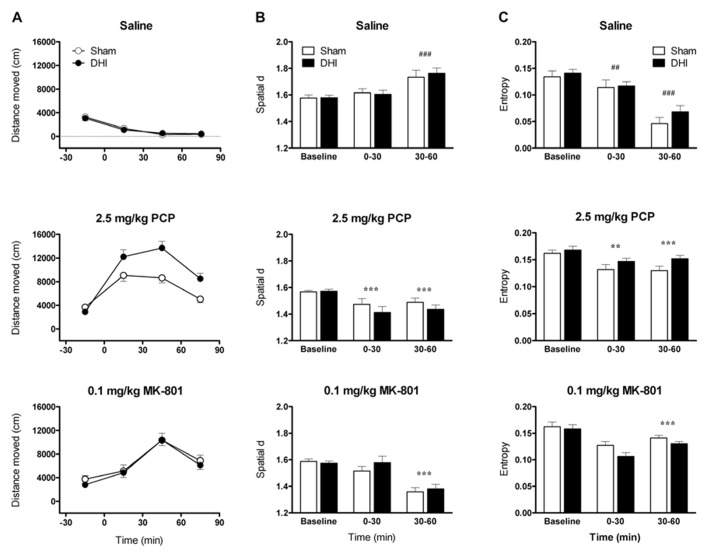
**Effect of 5,7-DHT-lesions targeting the dorsal hippocampus on qualitative aspects of locomotor hyperactivity induced by 2.5 mg/kg PCP and 0.1 mg/kg MK-801.** Panels show mean **(A)** distance moved (cm), **(B)** spatial d, and **(C)** entropy in 30 min intervals ± SEM for DHI (*n* = 12) and sham-operated rats (*n* = 11) treated with saline (top), 2.5 mg/kg PCP (middle), and 0.1 mg/kg MK-801(bottom). Total distance moved following 2.5 mg/kg PCP injection was, again, significantly higher in DHI rats compared to controls (see text for details). ^##^*p* < 0.01, ^###^*p* < 0.001 for comparison with baseline. **p < 0.01, ***p < 0.001 for comparison with saline in respective time block.

As observed in Experiment 1, average pre-injection distance moved did not differ between DHI and sham-operated rats in this experiment. Both groups habituated to the chambers with similar levels of activity (**Figure 2A**).

**FIGURE 3:**
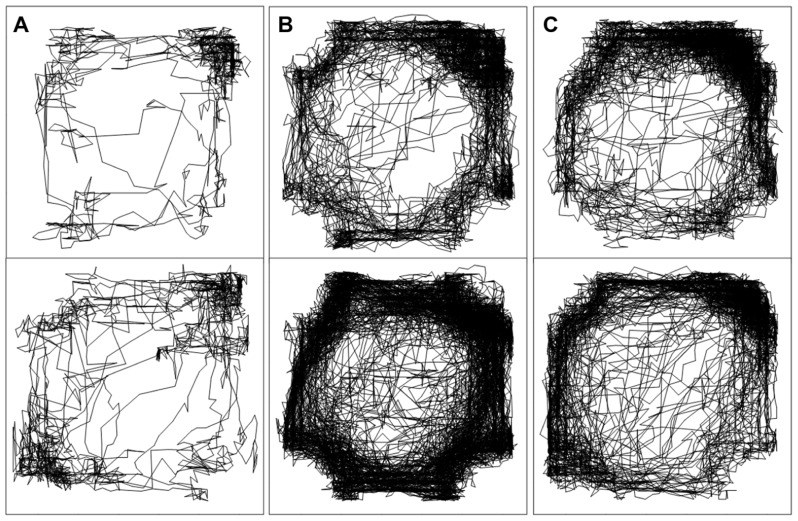
**Spatial patterns of locomotor hyperactivity shown by representative, individual sham-operated (top) and DHI (bottom) animals.** Plots show activity traces in the 60–90 min time block following treatment with **(A)** saline **(B)** 2.5 mg/kg PCP and **(C)** 0.1 mg MK-801. Enhanced PCP-induced locomotor hyperactivity in DHI rats is clearly depicted by the increased density of tracings in the chamber (panel **B**, bottom).

Analysis of variance of all post-injection distance moved data found that the effects of drug treatment were, again, dependent on lesion group (drug by group interaction: *F*_2,42_ = 6.3, *p* = 0.004; **Figure 2A**). As expected, total PCP-induced hyperactivity was greater in DHI rats than in sham-operated controls (51 ± 13% increase in total distance moved; main effect of group: *F*_1,21_ = 8.5, *p* = 0.008; drug by group interaction: *F*_1,21_ = 11.3, *p* = 0.003; **Figure 2A**, middle panel). In addition, MK-801 treatment caused a time-dependent increase in locomotor activity that was unaffected by dorsal hippocampus lesions (main effect of drug: *F*_1,21_ = 125.1, *p* < 0.001; drug by time interaction: *F*_2,42_ = 52.1, *p* < 0.001; **Figure 2A**, bottom panel). The lack of significant interactions between time and group in analyses of distance moved data for both compounds also corresponded with Experiment 1. Representative plots of post-injection activity are provided in **Figure 3**, in which enhanced PCP-induced hyperlocomotion in a DHI animal is clearly depicted (**Figure 3B**, bottom panel).

#### Spatial d

Assessment of average pre-injection spatial d revealed no difference in baseline levels between DHI and sham-operated rats (**Figure 2B**). After saline treatment, spatial d values increased over the course of the test sessions as the animals habituated to the chambers and made fewer smooth, linear exploratory movements (saline data only, comparison of all three time blocks: main effect of time, *F*_2,42_ = 18.9, *p* < 0.001; **Figure 2B**, top panel). Spatial d values exceeded baseline levels during the 30–60 min block (*F*_1,21_ = 27.7, *p* < 0.001) but not during the 0–30 min block.

Overall ANOVA of post-injection data found time-dependent, drug effects on spatial d that were unaffected by the lesions (main effect of drug: *F*_2,42_ = 45.3, *p* < 0.001; drug by time interaction: *F*_2,42_ = 46.4, *p* < 0.001). Compared to saline, both PCP and MK-801 treatments reduced spatial d in DHI and sham-operated rats (main effects of drug: PCP, *F*_1,21_ = 67.7, *p* < 0.001; MK-801, *F*_1,21_ = 61.4, *p* < 0.001), with more pronounced effects occurring in the 30–60 min time block (drug by time interactions: PCP, *F*_1,21_ = 9.5, *p* = 0.006; MK-801, *F*_1,21_ = 82.6, *p* < 0.001; **Figure 2B**). Further analyses for each time block revealed that PCP treatment reduced spatial d in both blocks (0-30 min, *F*_1,21_ = 23.5, *p* < 0.001; 30–60 min, *F*_1,21_ = 75.3, *p* < 0.001), whereas the effect of MK-801 treatment was significant in the 30–60 min interval only (0–30 min, *F*_1,21_ = 3.6, *p* = 0.071; 30–60 min, *F*_1,21_ = 135.5, *p* < 0.001). 5,7-DHT-lesions targeting the dorsal hippocampus, however, did not influence the changes in overall smoothness of the paths traveled following treatment with either compound.

#### Entropy

Analysis of mean pre-injection entropy data found no group differences, highlighting that the predictably of the paths traveled at baseline was unchanged by 5,7-DHT administration into the dorsal hippocampus (**Figure 2C**). In saline-treated animals, entropy values declined over the course of the session as the animals became habituated to the test chambers, indicating that the locomotor paths became progressively more repetitive (saline data only, comparison of all three time blocks: main effect of time, *F*_2,42_ = 51.6, *p* < 0.001; **Figure 2C**, top panel). Entropy levels were lower than baseline during both post-injection time blocks (0–30 min, *F*_1,21_ = 11.0, *p* = 0.003; 30–60 min, *F*_1,21_ = 103.5, *p* < 0.001).

Assessment of all post-injection data found an overall, time-dependent influence of drug treatment on entropy levels (main effect of drug: *F*_2,42_ = 34.6, *p* < 0.001; drug by time interaction: *F*_2,42_ = 35.5, *p* < 0.001; **Figure 2C**). Drug effects on entropy were further influenced by hippocampal 5,7-DHT lesions (drug by group interaction: *F*_2,42_ = 3.7, *p* = 0.032). PCP administration increased entropy compared to saline injection (main effect of drug: *F*_1,21_ = 51.1, *p* < 0.001), indicating that the animals traveled in less predictable manner around the chambers. While entropy levels following saline treatment decreased over time, after PCP injection entropy was elevated throughout the session (drug by time interaction: *F*_1,21_ = 28.5, *p* < 0.001; 0–30 min, *F*_1,21_ = 8.4, *p* = 0.009; 30–60 min, *F*_1,21_ = 65.1, *p* < 0.001). This effect was similar in sham-operated and DHI animals (**Figure 2C**, middle panel). Treatment with MK-801 also increased entropy compared to saline injection (main effect of drug: *F*_1,21_ = 36.5, *p* < 0.001) yet, similar to its effect on spatial d, this was significant only in the 30–60 min time block (drug by time interaction: *F*_1,21_ = 51.4, *p* < 0.001; 0–30 min, *F*_1,21_ = 0.024, *p* = 0.878; 30–60 min, *F*_1,21_ = 64.1, *p* < 0.001). Interestingly, compared to sham-operated controls, overall MK-801-induced enhancement of entropy was reduced in DHI rats (drug by group interaction: *F*_1,21_ = 4.6, *p* = 0.043; **Figure 2C**, bottom panel). Serotonergic lesions targeting the dorsal hippocampus, therefore, did not alter the random nature of locomotor paths traveled following PCP treatment, yet slightly reduced the extent to which MK-801 treatment increased this factor.

## DISCUSSION

There is considerable interest in the behavioral mechanism of action of PCP as it can produce a state in healthy humans analogous to symptoms in schizophrenia. Here we report that rats with 5,7-DHT-lesions targeting the dorsal hippocampus show potentiated locomotor hyperactivity following treatment with 0.5 or 2.5 mg/kg PCP, extending our earlier work by showing that the lesions are also sensitive to a five-fold lower dose (Kusljic and van den Buuse, 2004). Given the role of the hippocampus in spatial information processing, we anticipated that enhanced PCP responses in DHI rats would be associated with changes in the modulation of spatial d or entropy, yet analysis of behavioral patterns revealed no lesion effects on these variables at baseline or following PCP treatment. In contrast to PCP, DHI rats did not show a parallel enhancement of locomotor responses to MK-801, but rather a slight, but significant, reduction in MK-801-induced entropy. Thus, like lesions of serotonergic cell bodies in the MnR (Kusljic et al., 2005), 5,7-DHT-lesions targeting the dorsal hippocampus are sufficient to unmask functional differences between PCP and the more selective NMDA receptor antagonist, MK-801. Together with data from numerous locomotor activity experiments in 5,7-DHT-lesioned rats (see Adams et al., 2008 for review; Adams et al., 2009), these results indicate that serotonin projections from the MnR to the dorsal hippocampus are involved in the hyperlocomotor mechanism of action of the dissociative anesthetics, PCP and ketamine, as opposed to that of psychostimulants, like amphetamine, and in a manner seemingly independent of their ability to block NMDA receptors, or modulate spatial patterns of behavior. Like other reports (Snell et al., 1988; Hiramatsu et al., 1989; Rothman, 1994; Kapur and Seeman, 2002; Seeman et al., 2005; Seeman and Lasaga, 2005), our findings lend strength to the notion that the schizophrenomimetic effects of PCP and ketamine may not be due to NMDA receptor antagonism alone.

The differential effects of the lesions on PCP or MK-801-induced forward locomotion seem to hinge on impaired serotonergic tone in the dorsal hippocampus only, since rats with ventral hippocampus lesions in Experiment 1 showed no change in responses to either compound. In addition, dorsal hippocampus serotonin depletion appears robust in potentiating PCP responses regardless of the additional depletion observed in the ventral domain. In our early study, DHI rats showed no secondary lesion effects in the ventral hippocampus (Kusljic and van den Buuse, 2004); the asymmetric pattern of serotonin depletion observed in our DHI and VHI rats more recently has been discussed in detail elsewhere (Adams et al., 2009). However, with reduced serotonin levels in the whole hippocampus, we cannot definitively conclude that the behavioral changes are due to 5,7-DHT effects in the dorsal hippocampus only. Since entropy and spatial d was not assessed in VHI animals, it is possible that serotonin depletion in both hippocampal domains of DHI rats contributed to the reduction of MK-801-induced entropy. The ventral hippocampus also contains so-called “place cells,” involved in creating an internal representation of the environment (O’Keefe and Dostrovsky, 1971; Sweatt, 2004), suggesting that it, too, participates in spatial information processing (Moser and Moser, 1998). Nevertheless, the absence of corresponding functional changes in VHI rats in Experiment 1, together with our previous work (Kusljic and van den Buuse, 2004; Adams et al., 2009), suggests that serotonin depletion in the ventral hippocampus does not largely influence drug-induced hyperlocomotion.

Neither dorsal nor ventral hippocampal lesions were found to alter baseline locomotor behavior. Given that the expression of motor deficits after 6-hydroxydopamine-lesions depends on levels of dopamine depletion being >80–90% (Koob et al., 1981; Zigmond et al., 1990), it is possible that overt effects on baseline behavior were not seen due to insufficient levels of serotonin depletion. However, some of our previous cohorts have shown >80–90% depletion of hippocampal serotonin without showing alterations in baseline activity (Kusljic and van den Buuse, 2004; Adams et al., 2009). Even so, the utility of the 30 min habituation phase to assess baseline deficits may be questioned, as activity levels soon become negligible during this period making it difficult to evaluate any change. Indeed, others report that the extent of dorsal, but not ventral, hippocampal serotonin depletion correlates with the amount of activity displayed in the dark phase (Williams and Azmitia, 1981). Observing our lesioned animals in a novel open field, potentially in the dark phase, as well as incorporating assessments of vertical activity and grooming behavior, may provide a better appraisal of baseline lesion effects on motor behavior. In the present studies, as in our previous experiments, the expression of behavioral changes due to 5,7-DHT-lesions targeting the dorsal hippocampus was found to depend on pharmacological challenge (Adams et al., 2009; Adams and van den Buuse, 2011).

Phencyclidine has previously been shown to produce biphasic effects on spatial patterns of locomotion, generating smoother paths at 2.25 mg/kg (decreasing d) and reducing the smoothness of paths (increasing d) at higher doses (6.75, 10.125 mg/kg; Krebs-Thomson et al., 1998). Using the same analytical method, we similarly found that administration of 2.5 mg/kg PCP decreased d equally in both control and DHI rats. Interestingly, pre-treatment with a 5-HT_2A/2C_ agonist potentiated hyperlocomotion and further decreased spatial d in rats treated with 2.25 mg/kg PCP (Krebs-Thomson et al., 1998); together with the current data, one could speculate that 5-HT_2A/2C_ receptors in the dorsal hippocampus are involved in the former, but not the latter, effect. Finding that 0.1 mg/kg MK-801 also increases locomotion while producing smoother locomotor paths (decreasing d), and that both compounds reduce the predictability of the locomotor paths traveled (increasing entropy) is novel to this study. Hippocampal NMDA receptors are vital for spatial memory and information processing, with evidence indicating that they are necessary for the acquisition, or encoding, of spatial memory but not for retrieval (Nakazawa et al., 2004; Martin and Clark, 2007). One interpretation of the increase in entropy following MK-801 or PCP treatment is that NMDA receptor blockade impairs the animals’ memory of where they have previously been in the chamber, making them explore more randomly. In addition, 5,7-DHT-lesions targeting the dorsal hippocampus selectively reduced the ability of MK-801 to increase entropy, independent of its effect on locomotor activity. The mechanism underlying this more subtle effect of the lesions is unclear, but likely relates to a lesion-induced dysregulation of hippocampal NMDA receptors. Since PCP-induced entropy was not changed by the lesions, this could simply reflect the more potent and selective NMDA antagonist actions of MK-801.

Treatment with 0.1 mg/kg MK-801 and 2.5 mg/kg PCP produced equivalent levels of hyperlocomotion in control animals in both experiments. However, the maximal effect of MK-801 was seen between 30 and 60 min post-injection followed by a gradual decrease, while the peak PCP effect occurred earlier, between 15 and 30 min. The different times to onset of maximal effect of these agents corroborate previous studies (Ogren and Goldstein, 1994; Klamer et al., 2005), and may reflect differences in their temporal association to the NMDA receptor (Ogren and Goldstein, 1994). Indeed, MK-801 and PCP have similar volumes of distribution (Schwartz and Wasterlain, 1991; Shelnutt et al., 1999) and indexes of lipophilicity making them equally brain penetrant (Ault et al., 1995). In both experiments, however, the time to onset of PCP’s effects in DHI rats was more rapid, and the effect more vigorous and longer lasting, which is unlikely to arise from lesion-induced changes in pharmacokinetics. Instead, altered hyperlocomotor responses to PCP, and ketamine (Adams et al., 2009), in DHI rats appear to be a quantitative enhancement of the normal motor responses to these agents. In control animals, these compounds may activate serotonergic transmission in the dorsal hippocampus in a manner such that it inhibits their own effects. Accordingly, the absence of intact serotonergic tone in the dorsal hippocampus of DHI rats unmasks this self-activated, inhibitory mechanism. This could be significant to the mechanism of action of these dissociative compounds, as the lesion-induced enhancement was observed across all tested doses of PCP (0.5, 2.5 mg/kg) and ketamine (6.25, 12.5, 25 mg/kg; Adams et al., 2009).

Mechanisms within the dorsal hippocampus through which PCP and ketamine putatively activate serotonergic transmission could be pre- or post-synaptic, or a combination of both. As hypothesized earlier (Kusljic and van den Buuse, 2004), the effect could involve reduced PCP-induced serotonin release in the dorsal hippocampus (Martin et al., 1998a). Ketamine treatment increases extracellular serotonin levels in the ventral hippocampus (Lorrain et al., 2003), yet there is a lack of data regarding its effect in the dorsal domain. Serotonin release following treatment with these agents may result directly from SERT reuptake inhibition (Hori et al., 1996; Nishimura et al., 1998; Millan et al., 1999) or indirectly via glutamatergic disinhibition (Martin et al., 1998a), whereby preferential blockade of NMDA receptors on GABAergic interneurons by NMDA receptor antagonists “disinhibits” cortico-limbic circuits, causing the release of neurotransmitters (Olney et al., 1999). It follows, however, that MK-801 treatment would also disinhibit hippocampal circuits, and it was recently shown that local infusion of MK-801 increases extracellular serotonin levels in the dorsal hippocampus (Fallon et al., 2007). An alternative mechanism to explain the enhanced PCP responses may involve post-synaptic changes in serotonergic receptors. Autoradiography experiments revealed that local 5-HT_1A_ and 5-HT_2A_ receptor densities are unchanged by the lesions, yet there is a 70% increase in 5-HT_2C_ receptor densities in the dorsal hippocampus of DHI rats (Adams and van den Buuse, 2011). This finding is compelling because, unlike systemic administration, infusion of 5-HT_2C_ receptor agonists into the dorsal hippocampus stimulates locomotor activity (Takahashi et al., 2001; Stiedl et al., 2007). Thus, a simple explanation for our results would be a direct action by PCP on an upregulated 5-HT_2C_ receptor pool in the dorsal hippocampus of DHI rats. However, preliminary *in vitro *data indicate that PCP, ketamine or MK-801 do not directly bind to or activate the human 5-HT_2C_ receptor (unedited INI 5-HT_2C_ receptor isoform; Stewart and Christopoulos, Monash Institute of Pharmaceutical Sciences, Parkville, VIC, Australia, unpublished observations). While this does not exclude the possibility of a direct action on rat 5-HT_2C_ receptors, particularly in light of evidence that PCP and ketamine act directly on rat 5-HT_2A_ receptors (Nabeshima et al., 1988; Kapur and Seeman, 2002), this would have little relevance to humans. Additional locomotor behavioral experiments combining the local administration of selective ligands, such as for 5-HT_2_ receptor subtypes, into the dorsal hippocampus in conjunction with systemic PCP or MK-801 treatment are required. Finally, it is also possible that downstream alterations – either intrinsic or extrinsic to the hippocampus – might be involved in altered behavioral responses in DHI rats. It is clear that there are numerous mechanisms by which 5,7-DHT-lesions targeting the dorsal hippocampus could disclose differences in the hyperlocomotor effects of PCP and MK-801; the exact reasons remain speculative without further experiments.

Building on our previous 5,7-DHT-lesion studies in rats, our data highlight an important role for serotonin projections to the dorsal hippocampus, most likely from the MnR, in the mechanism of action of the dissociative anesthetics, PCP, and ketamine, but not that of MK-801. Given the direction and sensitivity of the behavioral change in dorsal hippocampus lesioned rats, in a normal state, these compounds may activate MnR-dorsal hippocampus serotonergic transmission in manner that subsequently serves to inhibit their net hyperlocomotor effects. The importance of clarifying the pharmacology of the “NMDA receptor antagonists” in the context of understanding their schizophrenogenic properties has been emphasized before (Klamer et al., 2005; Gilmour et al., 2009; Seillier and Giuffrida, 2009), with others finding that PCP and ketamine should not be used interchangeably (Gilmour et al., 2009). Understanding of how the “NMDA receptor antagonists” exert their hyperlocomotor effects in rodents is limited and, despite the seemingly analogous outward expression of locomotor hyperactivity they elicit, it is clear that the underlying neurochemical mechanisms are different. Elucidating these differences, particularly with neurotransmitter and brain region specificity, is important in the translation of preclinical research using these compounds in the context of schizophrenia.

## Conflict of Interest Statement

The authors declare that the research was conducted in the absence of any commercial or financial relationships that could be construed as a potential conflict of interest.
